# Modulation of thermometric performance of single-band-ratiometric luminescent thermometers based on luminescence of Nd^3+^ activated tetrafluorides by size modification

**DOI:** 10.1038/s41598-022-09912-4

**Published:** 2022-04-07

**Authors:** K. Trejgis, K. Ledwa, K. Maciejewska, L. Li, L. Marciniak

**Affiliations:** 1 Institute of Low Temperature and Structure Research, Polish Academy of Sciences, Okolna 2, 50-422 Wroclaw, Poland; 2 Hebei Key Laboratory of Optic-Electronic Information and Materials, College of Physics Science and Technology, Hebei University, Baoding, 071002 China

**Keywords:** Chemistry, Materials science, Optics and photonics

## Abstract

Due to a number of its advantages, luminescence thermometry has been a strongly developed strand of temperature metrology over a period of time. Although there are several different types of luminescent thermometers, recently attention has been focused on a new single-band ratiometric approach, which is based on the excited state absorption phenomenon. Nevertheless, since this process is nontrivial and has not been studied extensively in the context of thermometry to date, a number of studies are necessary to enable the intentional development of highly sensitive thermometers based on this method. One of the important aspects is to investigate the influence of material size and the associated occurrence of surface effects, which is considered in this work. In addition, the research in this paper has been extended to explore the aspect of host material composition. Accordingly, nanocrystals and microcrystals of *β*-NaYF_4_:2%Nd^3+^, *β*-NaGdF_4_:2%Nd^3+^, and LiGdF_4_:2%Nd^3+^ were investigated in this work. The influence of surface effects on thermometric parameters was proved, with special emphasis on the useful temperature range. Thus, by increasing the particle size, it was possible to intentionally extend the useful range by even more than 100 K.

## Introduction

Physical and chemical processes as well as biological phenomena are strongly dependent on temperature. Its changes are the key factor leading to control of these processes, therefore in many fields of science and industry it is indispensable to determine and control temperature with high precision, which often requires unconventional measurement methods tailored and optimized to the given application. One of the recent tendencies is the drive to development of remote and precise measurement methods in order to enable temperature determination even in harsh environments^1–3^, where contact methods are unreliable or even impossible to apply. One of the most commonly recognized techniques for remote temperature determination are thermovision cameras, however, the reliability of temperature readouts performed with them is questionable. Some limitations of thermovision cameras result from the so-called emissivity, which is an input parameter entered on the camera before the measurement. Depending on the deviation in the value of the input parameter, the temperature readout may be inaccurate by up to several hundred degrees^4^. Although for many materials the emissivity values are stabelled, in many cases substantial measurement errors occur. Furthermore, a thermal imaging camera can only measure the temperature of the first object encountered in the optical path, and thus has no ability to image the temperature in deeper layers, precluding its use in many situations. These limitations can be overcome by employing luminescence thermometry (LT), which enables determination of the temperature of both the surface and the interior of an object in a remote manner. Temperature is measured by analyzing thermally induced changes in one of the spectroscopic parameters of the phosphor, which acts as a thermometer^5–13^. Depending on the type of spectroscopic quantity being analyzed and the method of analysis, several types of luminescent thermometers are distinguished, of which, until recently the most extensively analyzed thermometers were those based on the ratio of the luminescence intensity of two emission bands^9,14–20^, however, several limitations of this technique have recently been highlighted^21–26^. One of the key drawbacks of this method is the need to spectrally resolve the emission bands involved in the ratiometric readout, which may often just not be simple, especially when the bands overlap each other. This strongly affects the reliability of the readout and is furthermore associated with high measurement costs or high complexity of the measurement system, since temperature 2D imaging requires hyperspectral tunable filters or switching between narrowband dichroic spectral filters. Even if the cost and complexity of the system are not an issue, a drastic factor affecting the precision of the temperature readout is modification of the shape of the emission spectrum associated with the dispersive character of the light absorption and scattering by the medium in which the thermometer is located. Hence, the ratio of the luminescence intensities of two bands can vary, making the definition of an universal calibration curve satisfied in any medium impossible. In an effort to overcome these factors and to improve the precision of the measurements, a new single-band ratiometric (SBR) approach has been developed that simultaneously provides a good readout quality guaranteed by maintaining ratiometricity (i.e., the presence of two signals to be compared against each other) while eliminating the influence of the dispersive dependence of the extinction coefficient by analysis of intensities in exactly the same spectral range for both considered signals. Temperature determination by this method is based on the analysis of the intensity ratio of a single emission band being excited by two wavelengths, where one pumping optical beam resonates with ground state absorption (GSA) like conventionally used excitation, and the other with excited state absorption (ESA)^13,21–29^. The high relative sensitivity of the thermometer based on this approach is due to the opposite nature of the thermal changes in emission intensity under these two excitation wavelength. With increasing temperature, the intensity of GSA-excited luminescence decreases due to various thermal luminescence quenching processes, of which, in the care of Ln^3+^ ions, multiphonon relaxation has a prominent role. In contrast to this, at low temperature the intensity of ESA-excited luminescence is negligibly small, while above a certain threshold temperature it starts to increase strongly with temperature. This is due to the fact that, according to the Boltzmann distribution, the thermal energy supplied to the system is one of the most effective channels of the population of the excited level from which the ESA process occurs. This opposing excitation wavelength-dependent thermal characteristic of luminescence intensity changes provides an opportunity to develop a new, efficient and highly sensitive tool for quantifying or imaging temperature.

As it was revealed in the studies reported recently, the thermometric parameters of an SBR thermometer depends on both the host material and the type and concentration of dopant ions^30,31^. First of all, a key factor in the ESA process is the probability of nonradiative transitions, which strongly depends on the energy difference between a given energy level and the next lower laying state, host phonon energies, and surface-related effects. One of the approaches to control the probability of nonradiative transitions may be through appropriate host selection. Among many different luminescent materials used for remote temperature determination, inorganic hosts doped with rare earth ions (RE^3+^) seem to be the most suitable. Although other materials may have better specific properties, e.g. quantum dots with higher luminosity or some biopolymers with higher biocompatibility than rare earth ion doped inorganic hosts, the latter are characterized by a balanced set of properties such as high chemical, mechanical and thermal stability, good photostability, high emission intensity, lack of photo blinking effect and low cytotoxicity which in general distinguish them from other materials^32–40^. One step to minimize the efficiency of nonradiative depopulation of excited states, according to the energy gap rule which determines the probability of nonradiative transitions, is to target materials with low energy host phonons^41–44^. Therefore, in order to reduce the influence of nonradiative processes on the excited level filling, hosts with low phonon energy, such as fluorides (phonon energy of about 350–450 cm^−1^)^45,46^, are preferred^47^. Of all the fluoride hosts, β-NaYF_4_ is the most common in the literature, however, the spectroscopic properties of the luminescent ions as well as the probability of the ESA process may strongly depend on the local symmetry of the ions, which can be modified by variations in the host material composition. Replacement of Y^3+^ sites in β-phase NaYF_4_ nanocrystals by Ln^3+^ ions can affect the unit cell volume, metal-oxide distances, and average crystallite size, and thus generate differences in spectroscopic properties. For instance, substitution of Y^3+^ with larger Gd^3+^ ions generates an expansion of the unit cell volume, and although *β*-NaYF_4_ and *β*-NaGdF_4_ adopt the same symmetry group ($P\overline{6}$ no.174)^48^ some small structural differences between them occur^49^. Significant changes in spectroscopic properties can also be generated by substitution Na^+^ ions with other alkali metal ions. A great example are Li^+^ ions, which are characterized by the smallest ionic radius in the group and, due to the reduced size, they generate a strong electric field around them causing deformation of anions, metal–oxygen distances, and symmetry. These features strongly distinguish lithium ions from other alkali metals, and in the fluoride host can strongly affect the luminescence properties of the Ln^3+^ dopant. The above information became the motivation to investigate the three *β*-NaYF_4_, *β*-NaGdF_4_ and LiGdF_4_ fluoride hosts for application in single-band ratiometric luminescence thermometry based on ESA phenomenon. Nevertheless, LiGdF_4_ nanocrystals are very rarely analyzed in general in the literature, which is due to the difficulty in synthesizing single-phase LiGdF_4_ nanocrystals^50,51^. Usually for light rare earth ions (lanthanides from La to Tb), LiF and REOF appear instead of LiREF_4_ particles. Actually, the limiting light lanthanide ions are Eu^3+^ ions for which a stable LiEuF_4_ structure has still been reported, however, for lighter ions this structure is rather absent^52^. One solution to stabilize the structure is doping with Y^3+^ ions. In the literature, one can find information proving an optimal amount of doping of 45 mol% Y^3+^ ions into the Gd^3+^ sites, resulting in the synthesis of single-phase tetragonal Li(Gd,Y)F_4_ nanocrystals without any phase impurities^51^.

The second origin of nonradiative transitions is related to effects at the particle surface. Due to the fact that the surface of materials is often strongly defected, the spectroscopic properties of ions on the surface may differ from their counterparts in the bulk part of the material^53–55^. The degree of surface ion contribution to the total emission spectrum changes with size and is more prominent in nanocrystals^14^. This is due to the fact that as the size is reduced, the ratio of ions on the surface to bulk ions increases. In addition, the surface ions being at the interface between the nanocrystal and the medium are strongly affected by the vibrational mods of the medium or ligands, which leads to depopulation of excited states. Therefore, investigating the effect of size on the intensity of ESA-induced luminescence may be a key factor in the development of highly sensitive single-band ratiometric luminescence thermometers.

One of the ideal candidates for single-band ratiometric luminescence thermometry applications are Nd^3+^ ions. This is primarily due to their energy level scheme. The distribution of energy levels allows for the occurrence of not only the ESA process, but also the (^4^F_3/2_; ^4^I_9/2_) → (^4^I_15/2_; ^4^I_15/2_) cross-relaxation (CR), which followed by nonradiative depopulation to lower ^4^I_J_ levels can provide an additional channel of the excited ^4^I_11/2_ level population, thus facilitating the occurrence of the ESA process. Moreover, a very characteristic feature of Nd^3+^ ions is the possibility of both their excitation and emission detection in the near infrared (NIR) region^56,57^, which is highly desirable from the perspective of biomedical applications^58^. We have recently demonstrated that by increasing the size of the YAG:Nd^3+^ particles, the usable temperature range of SBR thermometer can be significantly extanded^27^. Furthermore, a comparison of the relative sensitivity of YAG:Nd^3+^ with *β*-NaYF_4_:Nd^3+^ reveals significantly higher thermometric performance of the later one^59^. These facts became the motivation to extend our research to include an additional comparison of the properties of nano- and micromaterials. In this work, we undertook to investigate both the effect of size and host composition on the thermometric properties of SBR-based luminescence thermometers based on Nd^3+^ ion emission. Thus, nanocrystals and microcrystals of *β*-NaYF_4_, *β*-NaGdF_4_ and LiGdF_4_ doped with 2% of Nd^3+^ ions were investigated.

## Experimental

### Materials preparation

Although a high concentration of Nd^3+^ ions facilitates the occurrence of CR phenomenon, which simultaneously may be very beneficial for filling of the excited level from which the ESA process occurs, the high concentration of dopant ions may also carry other consequences. First of all, it may lead to quenching of the GSA-excited luminescence through multiphonon relaxation. In addition, a high concentrations of a dopant of Nd^3+^ ions can also lead to an expansion of the unit cell dimensions^25^, causing additional changes in the crystal symmetry, but can also induce undesirable effects of self-heating^60^. Therefore, considering these factors, the concentration analyzed in this work was limited to 2% of Nd^3+^ ions, which, as previously shown, is optimal^25^.

#### Nanocrystals

The nanocrystals were synthesized by thermal decomposition method in boiling oleic acid and octadecene acting as solvents^51,61^.

Neodymium(III) acetate hydrate ((CH_3_CO_2_)_3_Nd·3H_2_O with 99.9% purity), yttrium(III) acetate hydrate ((CH_3_CO_2_)_3_Y·3H_2_O with 99.9% purity), gadolinium(III) acetate hydrate ((CH_3_CO_2_)_3_Gd·3H_2_O with 99.9% purity), ammonium fluoride (NH_4_F of 98% purity), acetic acid (CH_3_CO_2_H of 99% purity), oleic acid (CH_3_(CH_2_)_7_CH=CH(CH_2_)_7_COOH of 90% purity) and 1-octadecene (CH_3_(CH_2_)_15_CH=CH_2_ with 90% purity) were purchased from Sigma Aldrich. Sodium hydroxide (NaOH with 99.8% purity), ethanol (C_2_H_5_OH, 96% pure p.a.), methanol (CH_3_OH, 99.8%), n-hexane (C_6_H_14_, pure p.a.) and chloroform (CHCl_3_, 98.5%) were purchased from POCH S.A. (Poland). Lithium hydroxide (LiOH anhydrous with 99% purity) were purchased from Pol-Aura. All of the chemical reagents were used as received without future purification.

#### ***β***-NaYF_4_:2%Nd^3+^ and ***β***-NaGdF_4_:2%Nd^3+^ nanocrystals^61^

Appropriate amounts of neodymium acetate and either yttrium acetate or gadolinium acetate depending on the host synthesized (NaYF_4_, NaGdF_4_) were placed in a 250 mL three-neck round-bottom flask along with 22.5 mL of octadecene and 9 mL of oleic acid. The solution was then magnetically stirred and heated slowly to 140 °C under vacuum conditions and further stirred at this temperature for 30 min to form Y(oleate)_3_ or Gd(oleate)_3_ and to remove residual water and oxygen. At the same time, 0.22222 g (6 mmol; molar ratio F^-^:RE^3+^ = 4) of ammonium fluoride and 0.14999 g (3.75 mmol; molar ratio Na^+^:RE^3+^ = 2.5) of sodium hydroxide were weighed into another vessel and 10 mL of methanol was added and magnetically stirred togheter. In the next step, the temperature of oleates was reduced to 50 °C and the atmosphere was changed from vacuum to a gentle flow of nitrogen. When the temperature of the solution reached 50 °C, a methanol solution of NaOH and NH_4_F was added quickly to the flask through the side neck and stirred under these conditions for 30 min. After this time, the temperature was increased to 85 °C and the conditions were changed to vacuum to completely evaporate the methanol from the reaction mixture. After the methanol evaporation the reaction temperature was increased to 300 °C with a minimum temperature rise rate of about 15 °C/min and maintained at this temperature for 60 min under the nitrogen flow. The final transparent dispersion was then cooled to room temperature. The NPs were precipitated by addition of ethanol and isolated by centrifugation at 10,000 rpm (22,360.0 rcf) for 10 min. For purification, the resulting pellet was dispersed in a minimal amount of *n*-hexane and precipitated again with excess ethanol. The UCNPs were isolated by centrifugation at 14,000 rpm (43,825.6 rcf) for 10 min. The final product stabilized with OA ligands was dispersed in 3.75 cm^3^ of chloroform (CHCl_3_). The concentration of the final products of NaYF_4_ and NaGdF_4_ was 20 mg/cm^3^.

#### Li(Y,Gd)F_4_:2%Nd^3+^ nanocrystals^51,62^

Appropriate amounts of neodymium acetate (2%), yttrium acetate (45%) and gadolinium acetate (53%) were placed in a 250 mL three-neck round-bottom flask along with 15.75 mL of octadecene and 15.75 mL of oleic acid. The solution was then magnetically stirred and heated slowly to 150 °C under vacuum conditions and further stirred at this temperature for 30 min to form Y(oleate)_3_ and Gd(oleate)_3_ and to remove residual water and oxygen. At the same time, 0.22222 g (6 mmol; molar ratio F^-^:RE^3+^ = 4) of ammonium fluoride and 0.08981 g (3.75 mmol; molar ratio Na^+^:RE^3+^ = 2.5) of lithium hydroxide were weighed into another vessel and 10 mL of methanol was added and magnetically stirred togheter. In the next step, the temperature of oleates was reduced to 50 °C and the atmosphere was changed from vacuum to a gentle flow of nitrogen. When the temperature of the solution reached 50 °C, a methanol solution of LiOH and NH_4_F was added quickly to the flask through the side neck and stirred under these conditions for 40 min. After this time, the temperature was increased to 85 °C and the conditions were changed to vacuum to completely evaporate the methanol from the reaction mixture. After the methanol evaporation the reaction temperature was increased to 320 °C with a minimum temperature rise rate of about 15 °C/min and maintained at this temperature for 90 min under the nitrogen flow. The final transparent dispersion was then cooled to room temperature. The NPs were precipitated by addition of ethanol and isolated by centrifugation at 10,000 rpm (22,360.0 rcf) for 10 min. For purification, the resulting pellet was dispersed in a minimal amount of *n*-hexane and precipitated again with excess ethanol. The UCNPs were isolated by centrifugation at 14,000 rpm (43,825.6 rcf) for 10 min. The final product stabilized with OA ligands was dispersed in 15 cm^3^ of chloroform (CHCl_3_). The concentration of the final product of Li(Y,Gd)F_4_ was 5 mg/cm^3^.

Samples for spectroscopic measurements were prepared by dropping 0.75 μL onto a glass slide and evaporating the chloroform.

#### Microcrystals

AREF_4_ microcrystals were synthesized by hydrothermal method.

#### ***β***-NaYF_4_:2%Nd^3+^ and ***β***-NaGdF_4_:2%Nd^3+^ microcrystals^63^

In a typical procedure of NaREF_4_ (RE: Y^3+^,Gd^3+^), 10 mL RE(NO_3_)_3_ (0.2 M) was poured into an 20 mL of aqueous solution containing 2 mmol of Na_3_C_6_H_5_O_7_ (molar ratio RE:citNa = 1). After vigorous stirring for 30 min, 25 mmol NaF (molar ratio F:RE = 12.5) was added into the above solution. After additional stirring for 15 min, the as-obtained mixed solution was transferred into a Teflon-lined autoclave and maintained at 180 °C for 24 h. As the autoclave was cooled naturally to room temperature, the precipitates were separated by centrifugation, washed with deionized water and acetone in sequence, and then dried in air.

#### Li(Y,Gd)F_4_:2%Nd^3+^ microcrystals^64^

In a typical procedure of LiGdF_4_, 3 mL of EDTA (0.5 M) was added to a mixture containing 3 mL of gadolinium nitrate (0.6 mmol, molar ratio EDTA:Ln = 0.9) and 10 mL of deionized water. The solution was then stirred for 30 min to form a chelated Gd-EDTA complex. Then, a solution of 8 mL (1.0 M) of NH_4_F and 4 mL (1.0 M, the molar ratio of F:Ln ~ 13) of LiF aqueous solutions were dropped into the chelated Gd-EDTA complex with thoroughly stirring. Then, the milky colloidal solution was transferred to a Teflon lined autoclave and heated at 200 °C for 36 h. After cooling to room temperature, the obtained product was collected by centrifuging and washed with water and acetone in sequence, and then dried in air.

### Characterization

**Figure 1:**
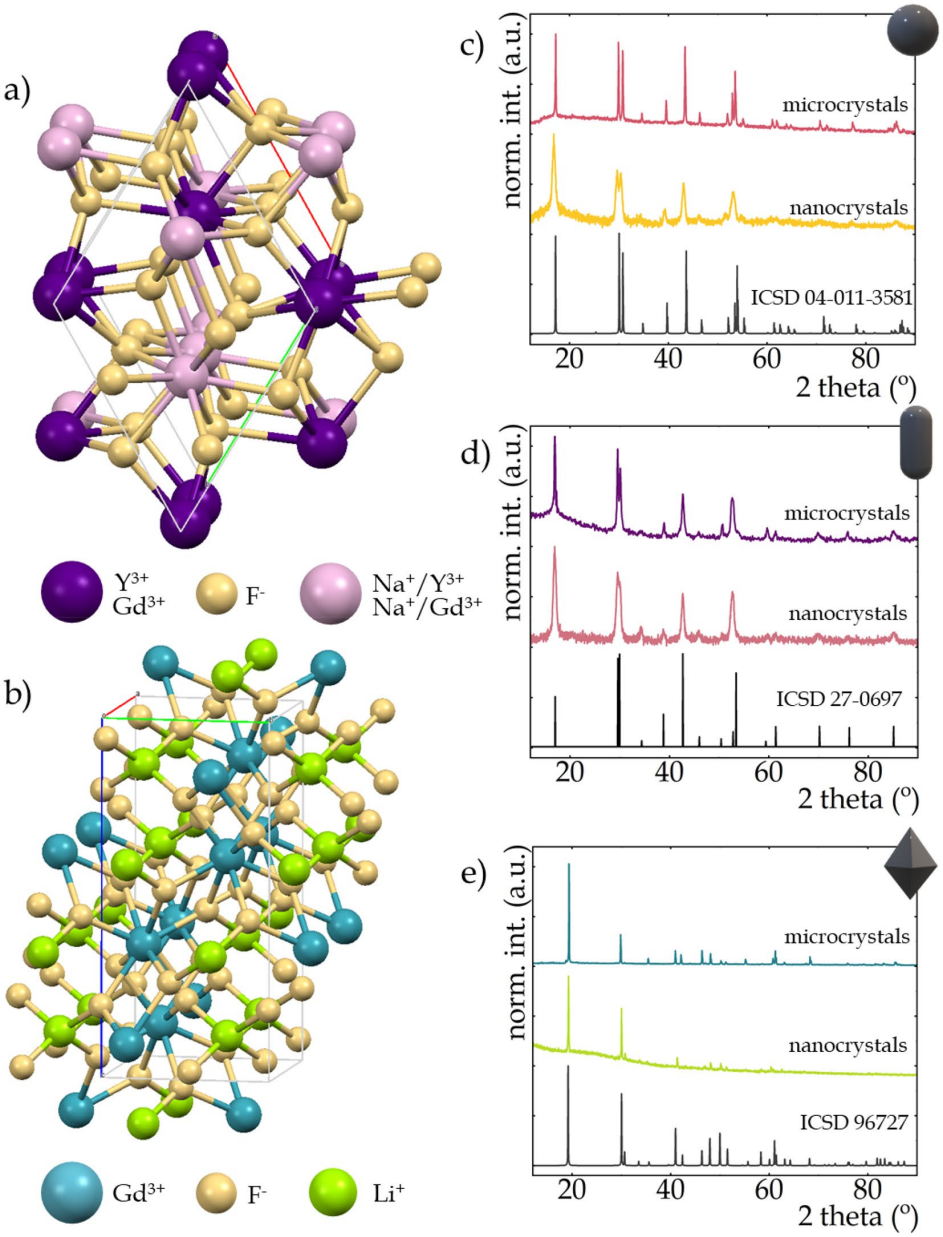
Visualization of Na^+^ and Y^3+^ or Gd^3+^ sites in the hexagonal NaYF_4_ or NaGdF_4_ structures (prepared using Mercury 4.2.0 software) (**a**) and of Li^+^ and Gd^3+^ sites in the tetragonal LiGdF_4_ structure (**b**); the Bragg reflections of *β*-NaYF_4_ (**c**), *β*-NaGdF_4_ (**d**) and LiGdF_4_ (**e**) nanocrystals and microcrystals.

Powder diffraction data were obtained using a PANalyticalX’Pert Pro diffractometer equipped with an Anton Paar TCU 1000 N Temperature Control Unit using Ni-filtered Cu Kα radiation (V = 40 kV, I = 30 mA). Transmission electron microscope (TEM) images were performed with the Philips CM-20 SuperTwin transmission electron microscope, operating at 160 kV. A drop of the suspension was put on a copper microscope grid covered with carbon. Morphology of the microcrystals was studied by scanning electron microscopy (SEM) using an FEI Nova NanoSEM 230 FE microscope equipped with an EDAX Genesis XM4 spectrometer. Prior to measurements, each sample was dispersed in CHCl_3_, and then a drop of the suspension was applied to a carbon stub and dried. Before the measurement, the sample was dried and purified in a H_2_/O_2_ plasma cleaner for 1 min. The excitation spectra and luminescence decay profiles were measured using the FLS1000 Fluorescence Spectrometer from Edinburgh Instruments equipped with 450 W Xenon lamp and μFlash lamp as an excitation sources and R5509-72 photomultiplier tube from Hamamatsu in nitrogen-flow cooled housing as a detector. To carry out the temperature measurement, the temperature of the sample was controlled using a THMS 600 heating–cooling stage from Linkam (0.1 °C temperature stability and 0.1 °C set point resolution). The emission spectra were measured using the 808 nm and 1060 nm excitation lines from laser diodes (LD) and a Silver-Nova Super Range TEC Spectrometer from Stellarnet (1 nm spectral resolution) as a detector. Visualizations of the fluorides structures (presented in Fig. 1a, b) were prepared using Mercury 4.2.0 software.

## Results and discussion

One of the key requirements of luminescence thermometry is a high luminescence efficiency, and in the case of the SBR approach it is additionally important to observe a high intensity of ESA-excited luminescence, which can be favorably influenced by interionic interactions via cross-relaxation that lead to an increased occupation of the excited state from which ESA occurs. Although two polymorphs of the NaYF_4_ structure are generally known, i.e. crystallographic α-cubic and β-hexagonal phases, the two aforementioned requirements appear to a greater degree in tetrafluorides crystallizing into the β-polymorph^65,66^, so the β-phase structures of NaYF_4_:2%Nd^3+^ and NaGdF_4_:2%Nd^3+^ are analyzed in this work. The β-phase crystallographic structure of NaYF_4_ (and NaGdF_4_) exhibits an ordered distribution of F^−^ ions accompanied by two cationic positions. One of them is fully loaded with Y^3+^ ions (or Gd^3+^ in the NaGdF_4_ matrix) and the other contains a mixed occupancy of Na^+^ and Y^3+^ (or Gd^3+^) ions (Fig. 1a). The unit cell parameters in the hexagonal phase of NaYF_4_ take the values *a* = *b* = 5.96 Å and *c* = 3.53 Å, while in NaGdF_4_ these parameters take the values *a* = *b* = 6.02 Å and *c* = 3.60 Å. This enlargement of the unit cell parameters observed for NaGdF_4_ is due to the larger ionic radius of Gd^3+^ ions (ionic radius of 0.938 Å^67^) relative to Y^3+^ ions (ionic radius of 0.900 Å^67^). In the case of the LiGdF_4_ host, a tetragonal structure is expected, however, due to a preference for the formation of LiF and GdOF structures during synthesis, there are some difficulties in synthesizing the single-phase tetragonal LiGdF_4_ nanocrystals. Therefore, to stabilize the structure to obtain nanocrystals with a single tetragonal phase (Fig. 1b), a Y^3+^ co-dopant was involved creating Li(Y,Gd)F_4_:2Nd^3+^. Unlike the *β*-NaYF_4_ and *β*-NaGdF_4_ hosts, which adopt the $P\overline{6}$(no.174) symmetry group^48^, LiGdF_4_ has the *I4*_*1*_*/a* (no.88) symmetry group^62,68^. In the LiGdF_4_ structure, Li^+^ and Gd^3+^ ions are fourfold and eightfold coordinated by F^-^ ions, respectively. The unit cell parameters in this material take the values of *a* = *b* = 4.96 Å and *c* = 10.93 Å. It follows that changing the host from *β*-NaYF_4_, *β*-NaGdF_4_ to Li(Y,Gd)F_4_ results in a significant increase in the unit cell volume from 109.1342 Å^3^, 114.0096 Å^3^ up to 269.0887 Å^3^, which may have further implications on the spectroscopic properties. X-ray diffractograms of nanocrystals and microcrystals and their corresponding reference patterns ICSD 04-011-3581, ICSD 27-0697 and ICSD 96727 successively for *β*-NaYF_4_, *β*-NaGdF_4_ and Li(Y,Gd)F_4_ structures are presented in Fig. 1c–e, respectively. Additional symbolism has been introduced in the upper corners of the corresponding diffractograms, where the sphere stands for *β*-NaYF_4_, the ellipsoid for *β*-NaGdF_4_, and the octahedron symbolizes the LiGdF_4_ structure. This symbolism will be retained in later sections of the manuscript to make the drawings clearer. The X-ray diffraction reflections of the synthesized materials correlate well with the reference patterns, which confirms that the phase purity of samples was not affected by particles size. It should be noted that the broadening of the Bragg reflections visible for the nanocrystals with respect to the standard pattern and reflections observed in the microcrystals results from the small size of nanocrystallites^69–71^.

**Figure 2:**
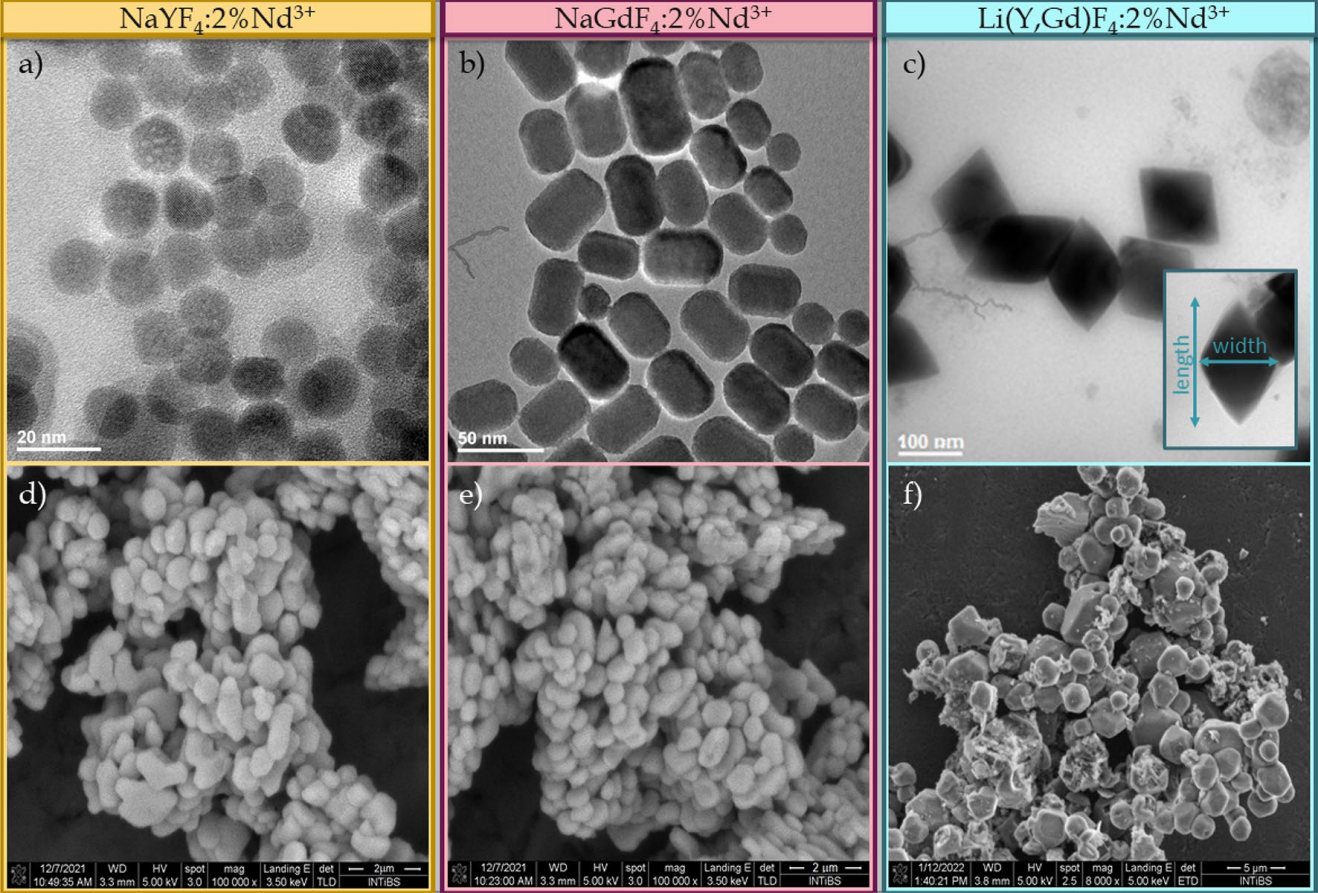
TEM images of *β*-NaYF_4_:Nd^3+^ (**a**), *β*-NaGdF_4_:Nd^3+^ (**b**) and LiGdF_4_:Nd^3+^ (**c**) nanocrystals and SEM images of *β*-NaYF_4_:Nd^3+^ (**d**), *β*-NaGdF_4_:Nd^3+^ (**e**) and LiGdF_4_:Nd^3+^ (**f**) microcrystals.

The small size of the nanocrystallites was confirmed by TEM images presented in Fig. 2a–c (for *β*-NaYF_4_:Nd^3+^, *β*-NaGdF_4_:Nd^3+^ and Li(Y,Gd)F_4_:Nd^3+^, respectively). The morphological analysis reveals that non-aggregated, homogeneous nanocrystals with a narrow size distribution were obtained. The *β*-NaYF_4_:Nd^3+^ nanocrystals exhibit a spherical shape with an average size determined by the Ferret’s method^72,73^ of about 14 nm ± 2 nm (Fig. 2a; Fig. S1a), while, as expected, the *β*-NaGdF_4_:Nd^3+^ nanoparticles assumed an elongated along one direction shape (Fig. 2b) with a larger size, i.e. 40 ± 10 nm in length and 28 ± 5 nm in width (marked in Fig. S1b in pinkish and purple, respectively). As previous studies by other research groups have shown, the different shape of these materials may be due to differences in atomic number and also ionic radius of RE^3+^ ions^49,74^, which affect the free energy of the system. The free energy dependence of the system shows that the rod-like NaREF_4_ particles are more stable than their spherical counterpart^75^, which is consistent with other finding that NaGdF_4_ in the hexagonal phase is more energetically stable than the NaYF_4_ counterpart in this phase^74^. For changes in the host of the alkali metal ion, the morphological changes are also observed. A bipyramidal shape is observed for Li(Y,Gd)F_4_:Nd^3+^ nanocrystals, which is characteristic for these materials obtained by thermal decomposition^76–78^ (Fig. 2c). The average sizes determined by Ferret's method were 170 ± 10 nm in length and 120 ± 10 nm in width (inset of Fig. 2c; Fig. S1c). The caveat here, however, should be that the size is somewhat beyond the realm of nanoparticles, which are generally considered to be particles up to 100 nm in size. Due to these larger sizes of Li(Y,Gd)F_4_ nanocrystals than of *β*-NaYF_4_ and *β*-NaGdF_4_, the broadening of the Bragg reflections for these nanocrystals seen in Fig. 1e is not as apparent as for the other nanocrystals analyzed (Fig. 1c, d). SEM images of the microcrystals revealed aggregated particles with an average size of about 1 μm for *β*-NaYF_4_:2%Nd^3+^ and *β*-NaGdF_4_: 2%Nd^3+^ phosphors (Fig. 2d, e, respectively), and larger LiGdF_4_:2%Nd^3+^ particles with size distributions ranging from 1 to nearly 5 µm (Fig. 2f).

**Figure 3:**
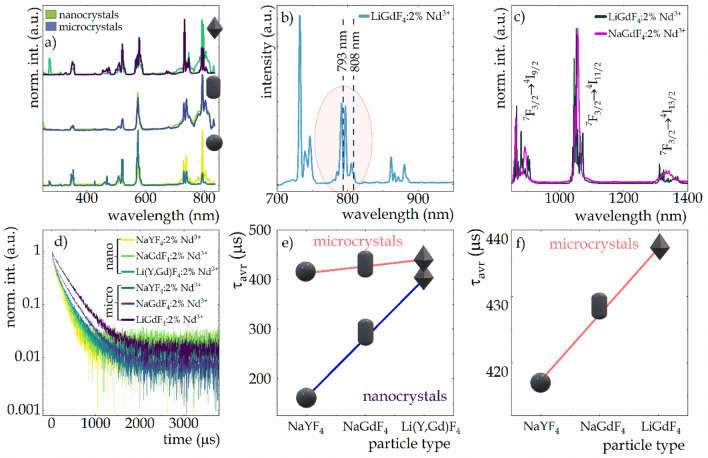
Comparison of excitation spectra of Nd^3+^ ions in three nanocrystalline and microcrystalline phosphors (**a**); excitation spectrum of Li(Y,Gd)F_4_:2%Nd^3+^ nanocrystals limited to the spectral range of 700–950 nm showing the shift of the excitation peak maxima with respect to the excitation wavelengths of 793 nm and 808 nm (**b**); comparison of emission spectra of *β*-NaGdF_4_:2%Nd^3+^ and Li(Y,Gd)F_4_:2%Nd^3+^ nanocrystals (**c**) impact of host composition and material size on luminescence decay curves (**d**) effect of host type on average decay times in nanocrystals and microcrystals (**e**) and in microcrystals shown in enlarged scale (**f**).

Trivalent Nd^3+^ ions are one of the most suitable ions for luminescence thermometry dedicated to biomedical applications, which is due to the occurrence of both emission bands (Fig. 3c) and excitation capabilities (Fig. 3a, b) in the infrared region. In general, the 793 nm excitation line enables the population of the emitting ^4^F_3/2_ state via the ^4^I_9/2_ → ^4^F_5/2_,^2^H_9/2_ absorption followed by nonradiative depopulation. For the excitation spectra of Nd^3+^ ions in the LiGdF_4_ host, there is a shift of the excitation bands relative to those in the NaYF_4_ matrix toward shorter wavelengths. As a result of this subtle blueshift, none of the commonly used IR laser diodes used to excite Nd^3+^ ions (i.e., 793 nm and 808 nm) at room temperature hit the maximum of the excitation bands, but only at its edge (Fig. 3b). At higher temperatures, due to electron–phonon coupling, this band may broaden, so that the absorption cross section at 793 nm or 808 nm may increase, which may affect the thermal dependence of the emission intensity excited at these wavelengths. As a consequence of the radiative depopulation of the ^4^F_3/2_ state to the ^4^I_9/2_, ^4^I_11/2_ and ^4^I_13/2_ levels, three characteristic Nd^3+^ bands at about 880 nm, 1050 nm and 1330 nm, respectively appear in the emission spectra (Fig. S2). In general, the luminescence of Nd^3+^ ions in the tetragonal LiGdF_4_ host exhibited much higher intensity, and the emission band splitting was more pronounced than in the hexagonal NaYF_4_ and NaGdF_4_ hosts (Fig. 3c; Fig. S2). This is due to the lower local symmetry around the Nd^3+^ ions in the tetragonal LiGdF_4_ crystal^79^ (eight-fold coordinated Gd^3+^ sites in the form of a square anti-prism with local *S*_*4*_ symmetry) than in the hexagonal counterpart^80^ (both RE^3+^ sites are nine-fold coordinated tri-capped trigonal prisms with local *C*_*3h*_ symmetry), so that the crystal field results in a more pronounced splitting of the Nd^3+^ ion multiplets into Stark levels^81^. Moreover, changing the local symmetry of the ion can also modify the luminescence decay times^82–84^ (Fig. 3d–f). As can be seen in Fig. 3d, lowering the symmetry results in this case in elongated decay times, which has already been noted in the literature^82^. Nevertheless, the observed trend of the radiative decay rate of 4f^n^ levels can also be analyzed in the framework of Judd–Ofelt theory. A starting point may be the consideration of the covalency of the material, which affects the magnitude of the Ω2 Judd–Ofelt parameter. It can be concluded that Li^+^ is a harder cation than Na^+^, which makes LiREF_4_ more ionic than its NaREF_4_ counterpart, which contributes to the lower value of the Ω2 Judd–Ofelt parameter^85^. According to the order Li < Na < K, the degree of covalency increases, causing the host stiffness to decrease, thus decreasing the values of the stiffness-dependent Ω4 and Ω6 parameters. The Ω4 and Ω6 parameters, on the other hand, according to the reduced matrix elements, dominate the Nd^3+^ optical transitions. As their values decrease from LiREF_4_ to *β*-NaREF_4_, the probability of radiative decay should also decrease in this order, resulting in the observation of longer luminescence decay times of Nd^3+^ in LiGdF_4_ host. Furthermore, a strong effect of particle size on the lifetime of the ^4^F_3/2_ emitting level was observed, with a more pronounced host effect manifested for nanocrystals, which may be due to the larger size of Li(Y,Gd)F_4_ nanoparticles than Na^+^ ion-containing counterparts (Fig. 2). Due to the fact that the decay profiles deviate from the exponential curve, the average lifetimes presented in Fig. 3e, f for nanocrystals and microcrystals, respectively, were calculated based on the double exponential profile according to the procedure described in the Supporting Information. In both nanocrystals and microcrystals, a similar trend in the effect of structure on the average decay times was observed, however, for microcrystals, the degree of change was significantly lower. With the change in host from *β*-NaYF_4_, *β*-NaGdF_4_ to LiGdF_4_, an elongation of the decay times from values of 166 μs, 291 μs to 408 μs in nanocrystals and 416 μs, 429 μs and 437 μs in microcrystals was evident. This trend may be related to the different local symmetry around the Nd^3+^ ions resulting from the increase in unit cell volume in *β*-NaYF_4_, *β*-NaGdF_4_, and LiGdF_4_, respectively, as described above. The shorter decay times in nanocrystals in respect to microcrystals are likely due to increased luminescence quenching associated with the material surface, which is highly defected. The spectroscopic properties of the ions located on the surface can differ significantly from their counterparts located in the bulk part of the materials^86^. A reduction in particle size increases the ratio of surface to bulk ions, thereby emphasizing the surface effect. In addition, surface ions are localized at the nanocrystal-medium interface, making them more strongly exposed to luminescence quenching by vibrational mods of the medium or ligand. Consequently, as a result of stronger surface-related quenching, the decay times in nanocrystals are shorter than in their microcrystalline counterparts, however, maintaining the trend related to the type of host.

**Figure 4:**
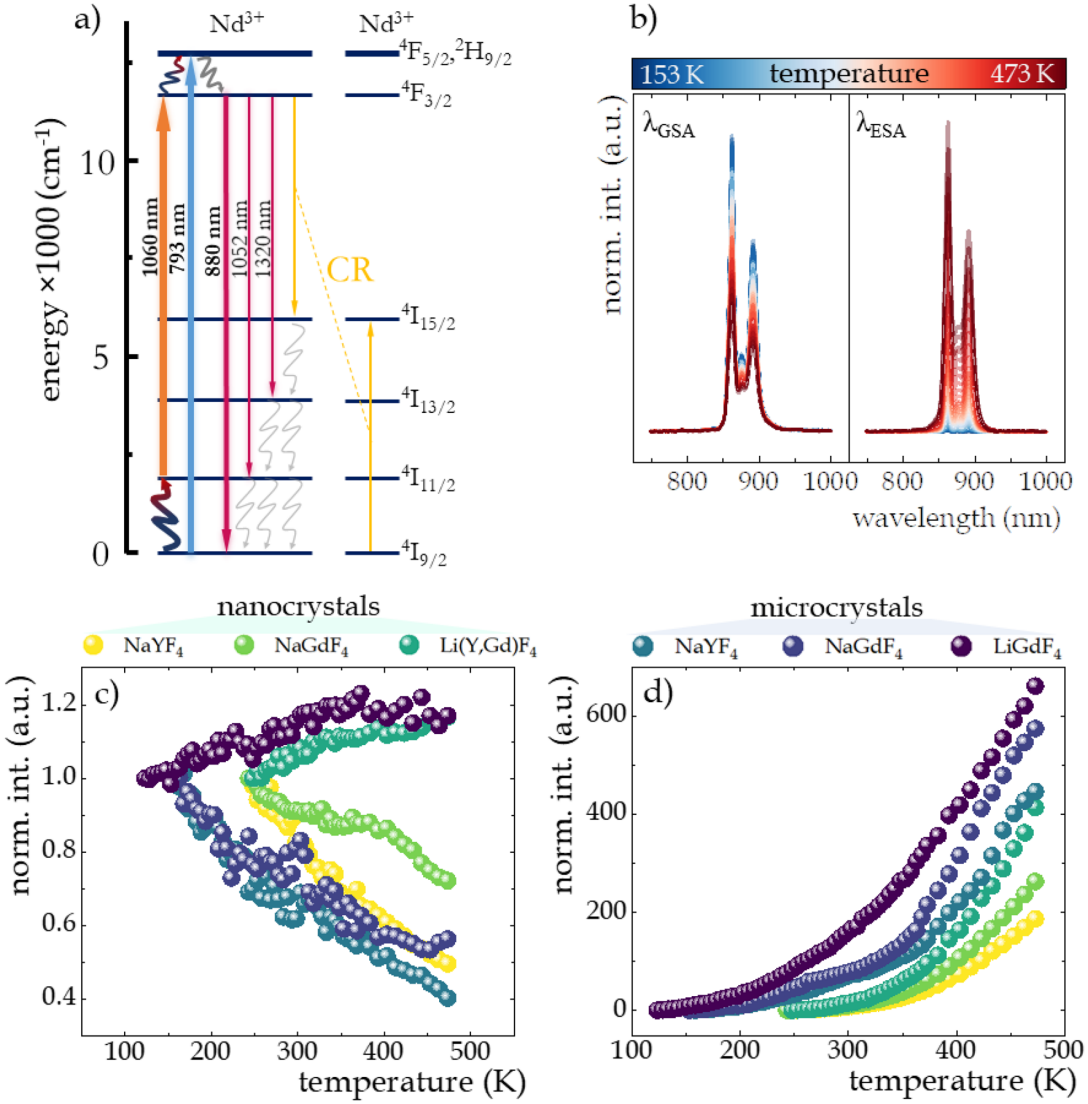
Simplified energy diagram of Nd^3+^ ions (**a**); representative thermal evolution of ^4^F_3/2_ → ^4^I_9/2_ emission band at 880 nm in *β*-NaYF_4_:2%Nd^3+^ upon λ_GSA_ = 793 nm and λ_ESA_ = 1060 nm excitations (**b**); the influence of the material size and host type on thermal evolution of normalized integral emission intensities of Nd^3+^ ions upon GSA (**c**) and ESA (**d**) excitation.

The electronic transitions between energy levels of Nd^3+^ leading to the generation of ^4^F_3/2_ → ^4^I_J_ luminescence are schematically described in Fig. 4a. When excited with a 793 nm beam matched to GSA, electrons from the ^4^I_9/2_ level are transferred to the ^4^F_5/2_, ^2^H_9/2_ level, which is then immediately followed by nonradiative depopulation to the metastable ^4^F_3/2_ state, from which radiative transitions occur. Although at low temperature this type of excitation is dominant, at high temperatures excited state absorption (ESA) can also occur. This is due to the fact that the energy distance between the ^4^I_9/2_ and ^4^I_11/2_ levels of the ground multiplet is on the order of 2000 cm^−1^, which can be easily overcome by the thermal energy supplied to the system, which promotes some electrons from the ground state to the ^4^I_11/2_ level. These can then be excited with a wavelength of 1060 nm to the ^4^F_3/2_ level. Although such a Boltzmann distribution-consistent process of thermalization of the excited level with electrons from the ground level is the dominant and key process of filling of the ^4^I_11/2_ level, other processes can also occur, both of the population e.g. via (^4^F_3/2_; ^4^I_9/2_) → (^4^I_15/2_; ^4^I_15/2_) cross-relaxation (yellow arrows in Fig. 4a) followed by nonradiative transition to ^4^I_11/2_ state (grayish wavy arrows in Fig. 4a), or as a result of ^4^F_3/2_ → ^4^I_11/2_ emission transitions, due to the nearly 60% luminescence branching ratio corresponding to this transition, but also depopulation processes related to surface effects. For nanostructures, the ratio of ions on the surface of the phosphor relative to those in the volume part is larger and increases with size reduction, resulting in luminescent properties more strongly affected by interactions with functional groups of the medium or with ligands attached to the surface of the nanocrystals^87^. Because the energy distance between the ^4^I_11/2_ and ^4^I_9/2_ levels of 2000 cm^−1^ is less than the energy of one vibrational mode of the OH^-^ groups^88^, surface effects can strongly contribute to the depopulation of the excited level. Although as a result of the excitation of the ^4^F_3/2_ level and its subsequent radiative depopulation several emission bands at 880 nm, 1058 nm and 1330 nm are possible, of which the band at 1058 nm is characterized by the highest emission intensity, in further studies of temperature-dependent spectroscopic properties we focus our attention on the band at 880 nm (^4^F_3/2_ → ^4^I_9/2_) since the band at 1060 nm coincides with the wavelength matched to the ESA excitation making its detection impossible. To illustrate the influence of temperature change on the emission intensity of Nd^3+^ ions, the thermal evolution of the emission spectra of representative *β*-NaYF_4_:2%Nd^3+^ nanocrystals limited to the emission band at 880 nm associated with the ^4^F_3/2_ → ^4^I_9/2_ electronic transition (bold pink arrow in Fig. 4a) under both GSA and ESA excitation conditions are presented on the left and right sides of Fig. 4b, respectively. The clearly opposite direction of thermally induced changes in luminescence intensity of this band upon ESA versus GSA excitation has been evidenced. A qualitative comparison between all analyzed nanocrystals and microcrystals is provided by the integral emission intensities under GSA (Fig. 4c) and ESA (Fig. 4d) excitations. As expected, in both nanocrystals and microcrystals of *β*-NaYF_4_:2%Nd^3+^ and *β*-NaGdF_4_:2%Nd^3+^, with increasing temperature, the intensity of GSA-excited luminescence decreased due to multiphonon relaxation. On the contrary, the intensity of GSA-induced emission of Nd^3+^ ions in the LiGdF_4_ host gradually and gently increased by around 20% with increasing temperature in the 123–493 K range, regardless of the material size (Fig. 4c). The observed increase in the intensity of GSA-excited luminescence is due to the thermally induced broadening of the ^4^I_9/2_ → ^4^F_5/2_,^2^H_9/2_ excitation band. On the other hand, although at low temperatures the intensity of ESA-induced luminescence is low, it surges with increasing temperature (Fig. 4d). Although this trend is preserved for all materials analyzed, the growth rate strongly depends on both host type and crystal size. In both nanocrystals and microcrystals, the rate of luminescence intensity enhancement with temperature increases with the host switching in the order NaYF_4_:2%Nd^3+^  < NaGdF_4_:2%Nd^3+^  < LiGdF_4_:2%Nd^3+^, whereby in microcrystals the rate of change is significantly higher. In nanocrystals, the increase in intensity at 473 K relative to the intensity at the initial temperature at which the ESA process began to occur was about 190, 260, and 400 fold in *β*-NaYF_4_:2%Nd^3+^, *β*-NaGdF_4_:2%Nd^3+^ and LiGdF_4_:2%Nd^3+^ nanocrystals, respectively. In microcrystals, these values nearly doubled reaching 450, 575, and 600 fold growth for *β*-NaYF_4_:2%Nd^3+^, *β*-NaGdF_4_:2%Nd^3+^ and LiGdF_4_:2%Nd^3+^ microcrystals, respectively. Moreover, the threshold temperatures in microcrystals can be significantly shifted towards lower temperatures, up to 123 K (in LiGdF_4_:2%Nd^3+^) relative to the values obtained in nanocrystals (about 240–250 K ). Both the lower threshold temperatures and the stronger increase in the intensity of ESA-induced luminescence observed in microcrystals are due to the smaller influence of the surface-related quenching of the ^4^I_11/2_ level population than in nanocrystals and the increase in the crystalinity of the microcrystals. Furthermore, in order to determine the thermal stability, thermally induced changes in luminescence intensity were investigated for 10 heating–cooling cycles, confirming very high repeatability of the changes observed (Fig. S4).

Such different thermal dependence of the emission band of Nd^3+^ ions localized at 880 nm on the excitation wavelength provided the opportunity to develop a single-band radiometric luminescence thermometer in which the temperature-dependent parameter is determined by the luminescence intensity ratio (LIR) defined as follows:

1 $$LIR = \frac{{\int_{855}^{870} {^{4} {\text{F}}_{3/2} \to^{4} {\text{I}}_{9/2} (ESA){\text{d}}\lambda } }}{{\int_{855}^{870} {^{4} {\text{F}}_{3/2} \to^{4} {\text{I}}_{9/2} (GSA){\text{d}}\lambda } }}$$

**Figure 5:**
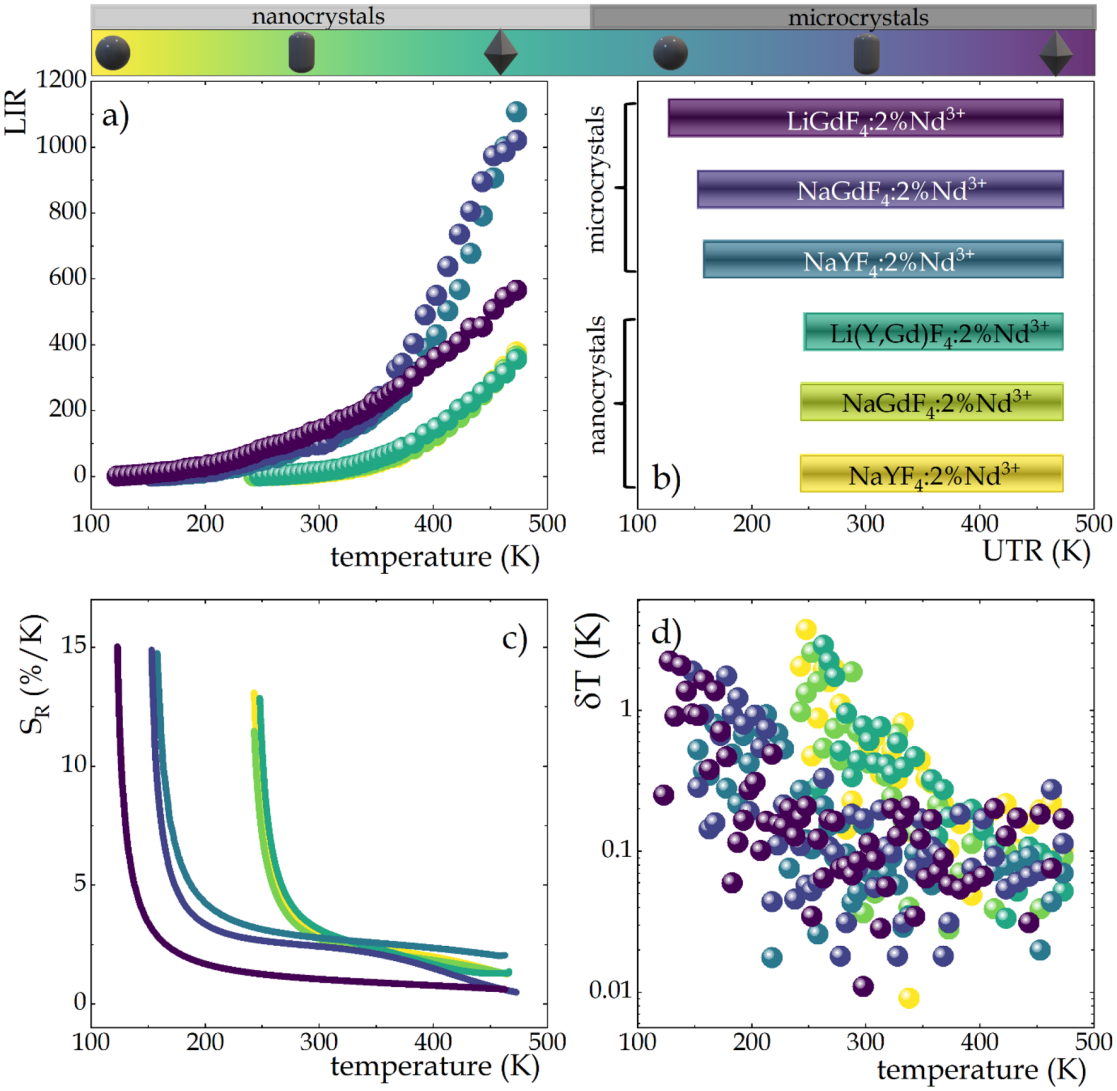
The temperature dependent LIR values of all analyzed materials doped with Nd^3+^ ions (**a**); influence of the host type and crystal size on the useful temperature range (**b**); the relative sensitivities of SBR LTs (**c**) and corresponding temperature resolutions (**d**).

In general, due to the greater susceptibility of ESA-excited luminescence to temperature changes, the nature of LIR changes is mainly determined by the trend of integral emission intensities upon ESA excitation, however, a strong deviation from this trend can be observed particularly for LiGdF_4_:2%Nd^3+^ microcrystals (Fig. 5a). Although the integral intensities of ESA-excited luminescence exhibited clear trends related to both host type and crystal size, the temperature dependence of the LIR parameter in the nanocrystals is almost independent of host type and at 473 K the LIR value in all analyzed nanocrystals reaches about 350 fold increase over the value at initial temperature. The observed lack of the effect of host type is due to the progressively weaker quenching of GSA-induced luminescence when the phosphor was changed in the order *β*-NaYF_4_:2%Nd^3+^, *β*-NaGdF_4_:2%Nd^3+^ and LiGdF_4_:2%Nd^3+^ (Fig. 4c). The efficient nonradiative depopulation of the ^4^F_3/2_ emitting state is reflected both in a lower rate of thermal enhancement of the ESA-excited luminescence and in an increase in the quenching rate of GSA-excited emission. The most noticeable changes are found in the thermal evolution of LIR of the LiGdF_4_:2%Nd^3+^ microcrystals, for which, although there is the highest integral intensity of ESA-induced luminescence, the LIR value increases only about 560-fold with respect to the value at the initial temperature, which is nearly twice weaker than in the other microcrystals (*β*-NaYF_4_:2%Nd^3+^, *β*-NaGdF_4_:2%Nd^3+^), where there is about 1100-fold enhancement of the LIR value with respect to the initial value. Similarly to the nanocrystals, in the case of the microcrystalline fluorides the host effect in not evident. Obviously, in the case of the LiGdF_4_:2%Nd^3+^ microcrystals, a thermal dependence different from that of other fluorides can be found, which results from the increasing with temperature the intensity of the GSA-induced luminescence. Furthermore, a threshold temperature above which the ESA process occurs and thus an increase in LIR is observed is clearly evident here. It results from the competing processes of thermal population and surface-related depopulation of the excited level and is reflected in the useful temperature range (UTR; Fig. 5b). Due to the fact that surface-related quenching effects are much stronger in nanocrystals than in larger structures, the useful temperature range in nanocrystals is significantly narrower and equals 230 K (243–473 K), while in LiGdF_4_:2%Nd^3+^ microcrystals it is widened by more than 100 degrees and equals 350 K (123–473 K).

The ability of luminescent thermometers can be quantified by the standardized parameter of relative sensitivity to temperature changes, determined as follows:

2 $$S_{R} = \frac{1}{LIR} \cdot \frac{\Delta LIR}{{\Delta T}} \cdot 100\%$$

where ∆LIR represents the change of LIR value in response the change of temperature ∆T. Regardless of the host type, in nanocrystals the maximum values of relative sensitivity were close to 13%/K while in microcrystals its values reached up to 15%/K (Fig. 5c). For all thermometers analyzed, the maximum sensitivity was obtained at low temperatures of the UTR corresponding to a particular sample. However, in this temperature range, the signal-to-noise ratio of ESA-excited luminescence is small, which reduces the reliability of the results. As the temperature increased, the relative sensitivity decreased, however, at room temperature (298 K) it still exceeded 1%/K. These results clearly indicate that by changing the size of the phosphor particles, it is possible to develop a luminescent thermometer of remarkably high relative sensitivity over a predefined temperature range. Based on such high sensitivities, temperature resolutions were determined to establish the ability of the analyzed SBR-based luminescent thermometers to determine temperature. Their thermal evolution was determined by the following equation:

3 $$\delta T = \frac{1}{{S_{R} }} \cdot \frac{\delta LIR}{{LIR}}$$

where

4 $$\frac{\delta LIR}{LIR}=\sqrt{{\left(\frac{\Delta {I}_{ESA}}{{I}_{ESA}}\right)}^{2}+{\left(\frac{\Delta {I}_{GSA}}{{I}_{GSA}}\right)}^{2}}$$

As can be seen in Fig. 5d, for both sizes of all analyzed materials, there is a large scatter of temperature resolution values in the low temperature range of the UTR corresponding to each thermometer. At low temperatures, in nanocrystals the resolution values are even above 3 K and in microcrystals above 2 K. The reason for the weak temperature resolutions in these temperature ranges is probably due to the relatively large error resulting from the low signal-to-noise ratio of the ESA-excited luminescence. In nanocrystals, temperature resolution values below 0.2 K were only achieved at temperatures above 360 K, whereas in microcrystals, this temperature was reduced by 130 K and temperature resolutions < 0.2 K were already achieved at 230 K. Based on the above results, it can be concluded that the local symmetry around the emitting ions affects the thermometric parameters of SBR-based luminescent thermometers only slightly, while a significant size change, as in the case of nanocrystals and microcrystals, can greatly affect both the useful temperature range, the relative sensitivity and, most importantly, the temperature resolution. As a result of surface interactions occurring more strongly in nanocrystals, the thermometric performance of such luminescent nanothermometers is weakened. Moreover, comparison with the previously reported results for YAG:Nd^3+^ (nanocrystals, microcrystals and optical ceramics) reveals that host material with lower phonon energies, e.g. fluorides, are desirable to obtain a luminescent thermometer with high thermometric performance^27^. A practical implementation of such a temperature readout of the SBR luminescent thermometer can be based on two excitation radiation sources fed by a phase-shifted rectangular electrical signal, as already presented in our recent work^89^. In this way, each wavelength would alternately excite the nanocrystals, and the luminescent signal could be recorded by a detection system in the form of camera with a bandpass filter. However, being aware that measurement in a medium other than air with a dispersive dependence of the extinction coefficient can significantly complicate the measurement, this approach should be verified experimentally.

## Conclusions

In this work, the impact of particle size and associated surface effects and matrix composition on the thermometric properties of single-band ratiometric luminescent thermometers based on Nd^3+^ ion emission in fluoride hosts were investigated. *β*-NaYF_4_:2%Nd^3+^, *β*-NaGdF_4_:Nd^3+^ and LiGdF_4_:2%Nd^3+^ phosphors in the form of nanocrystals and microcrystals were examined. In order to define the temperature-dependent single-band ratiometric LIR parameter based on the emission intensity at 880 nm (^4^F_3/2_ → ^4^I_9/2_ electron transition), thermometric studies were performed using two excitation wavelengths corresponding to absorption from the ground state (λ_EXC_ = 793 nm) and from the excited state (λ_EXC_ = 1060 nm). A strong temperature effect on the luminescence intensity was observed and explained, and the nature of the changes strongly depended on the excitation wavelength. In general, the thermally induced multiphoton nonradiative quenching of the ^4^F_3/2_ level results in a decrease in the intensity of the GSA-excited luminescence, whereas due to the thermal population of the ^4^I_11/2_ excited state, the intensity of the ESA-excited luminescence surged with increasing temperature. The determined temperature-dependent LIR parameter showed very pronounced changes with increasing temperature, and the rate of change depended on both the phosphor composition and the particle size. In the case of nanocrystals, the observed changes were much weaker than in the microcrystalline counterparts, which is due to the fact that a large ratio of ions on the surface relative to the total number of ions is present in nanocrystals, and their luminescence is strongly affected by surface interactions. As a result of interactions with ligands, or the medium, there may be surface-related nonradiative quenching effects on the occupancy of energy levels, in particular the ^4^I_11/2_ level from which ESA occurs. As a result, in nanocrystals the intensity of ESA-induced luminescence is lower than in larger particles, and the thermal energy required for the ESA process to occur is significantly higher. The high threshold energy required in nanocrystals for which the ESA process occurs is reflected in a much narrower useful temperature range than for microcrystals. As a result of the transition from nanocrystals to microcrystals, the useful temperature range can be intentionally extended. Although relative sensitivities are highest at low temperatures and reach over ten percent, due to the small signal-to-noise ratio of ESA-excited luminescence, results in this temperature range are subject to large error, which is represented by poor temperature resolution on the order of a few Kelvin degrees and should be excluded, further narrowing the operating temperature range of a given thermometer. Based on the temperature resolution results, it can be concluded that reliable results allowing ẟT < 0.2 K in nanocrystals occur only at temperatures higher than 350 K, while using microcrystals for single-band ratiometric thermometry, this range can be intentionally extended by more than 100 K (ẟT < 0.2 K in the temperature range 230–473 K). Furthermore, we find that the host composition only slightly affects thermometric parameters such as sensitivity, UTR, and temperature resolution.

## Supplementary Information

Supplementary Information.
