# Author Correction: Modulation of thermometric performance of single-band-ratiometric luminescent thermometers based on luminescence of Nd^3+^ activated tetrafluorides by size modification

**DOI:** 10.1038/s41598-022-11989-w

**Published:** 2022-05-09

**Authors:** K. Trejgis, K. Ledwa, K. Maciejewska, L. Li, L. Marciniak

**Affiliations:** 1 Institute of Low Temperature and Structure Research, Polish Academy of Sciences, Okolna 2, 50-422 Wroclaw, Poland; 2 Hebei Key Laboratory of Optic-Electronic Information and Materials, College of Physics Science and Technology, Hebei University, Baoding, 071002 China

Correction to: *Scientific Reports* 10.1038/s41598-022-09912-4, published online 07 April 2022

The Acknowledgements section in the original version of this Article was incomplete.

“This work was supported by National Science Center Poland (NCN) under Project No. UMO-2019/33/N/ST5/00011.”

now reads:

“This work was supported by National Science Center Poland (NCN) under Project No. UMO-2019/33/N/ST5/00011. Karolina Trejgis would further like to thank the Foundation for Polish Science (FNP) for its support.”

The original Article has been corrected.

