# Supplementary material for: Modulation of thermometric performance of single-band-ratiometric luminescent thermometers based on luminescence of Nd3+ activated tetrafluorides by size modification
Source: Sci Rep. 2022 Apr 7;12:5847. doi: 10.1038/s41598-022-09912-4 (PMC8989989; doi:10.1038/s41598-022-09912-4)
Supplement: Supplementary file 1 — Supplementary Information. [file 41598_2022_9912_MOESM1_ESM.docx]

**Supporting Information**

**Modulation of thermometric performance of Single-Band-Ratiometric Luminescent Thermometers based on luminescence of Nd^3+^ activated tetrafluorides by size modification**

K. Trejgis^1*^, K. Ledwa^1^, K.Maciejewska^1^, L. Li^2^, L. Marciniak^1*^

^1^Institute of Low Temperature and Structure Research, Polish Academy of Sciences, Okolna 2, 50-422 Wroclaw, Poland

^2^ Hebei Key Laboratory of Optic-electronic Information and Materials, College of Physics Science and Technology, Hebei University, Baoding 071002, China

* corresponding author: *[k.trejgis@intibs.pl](mailto:k.trejgis@intibs.pl)*, [*l.marciniak@intibs.p*](mailto:l.marciniak@intibs.p)*l*

KEYWORDS: SBR approach, luminescence thermometry, fluorides, neodymium, size effect


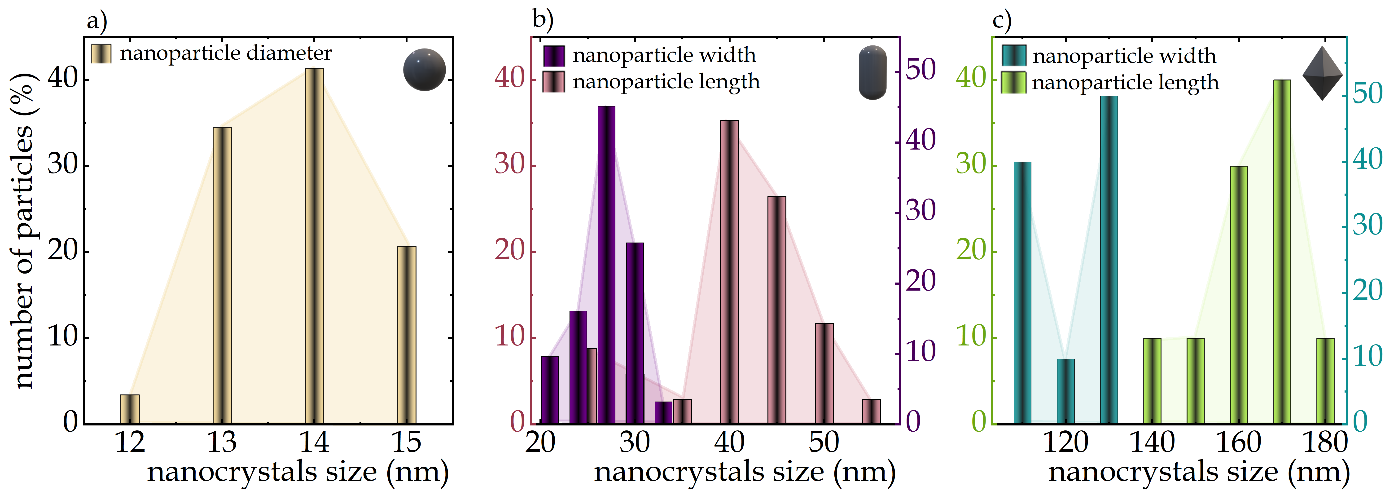


**Figure S1**. Size distributions of NaYF_4_:2%Nd^3+^ (a), NaGdF_4_:2%Nd^3+^ (b) and LiGdF_4_:2%Nd^3+^ (c) nanocrystals determined from TEM images using Feret's method.


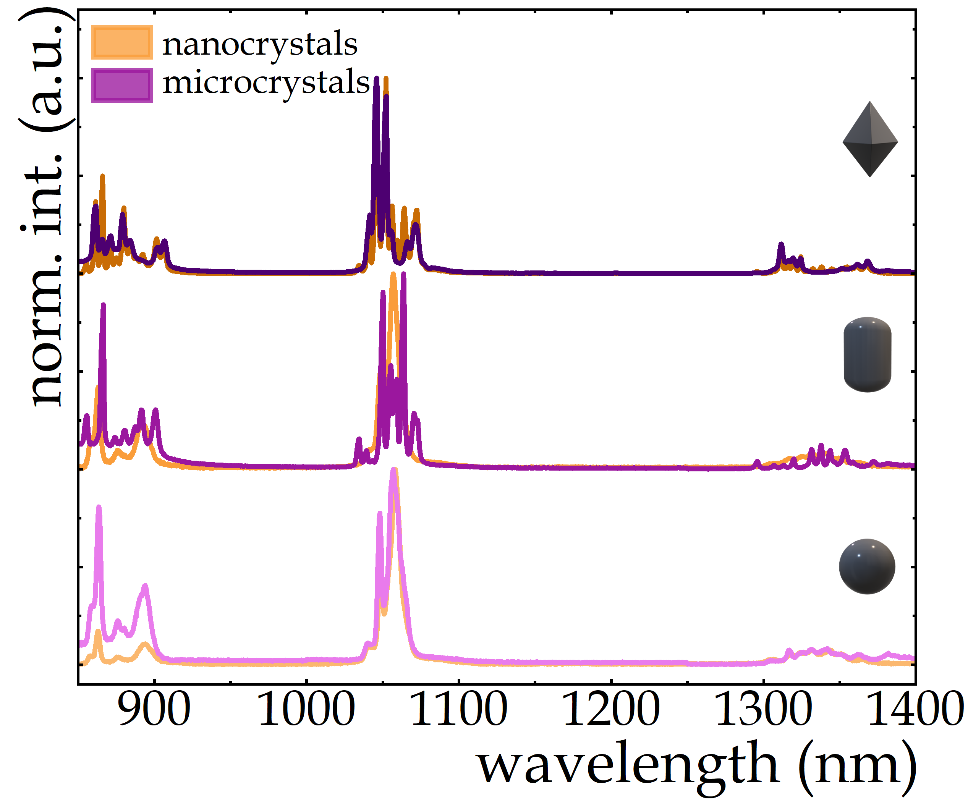


**Figure S2**. Comparison of emission spectra of Nd^3+^ ions in three nanocrystalline and microcrystalline phosphors.

Average luminescence lifetimes presented in inset of Fig. 3d were calculated using the following equation:

$\tau_{avr}=\frac{A_{1}\tau_{1}^{2}+A_{2}\tau_{2}^{2}}{A_{1}\tau_{1}+A_{2}\tau_{2}}$ (eq.S1)

Where all the parameters are obtained from the fitting of the experimental decay profile with double exponential function:

$y=A_{1}\cdot e^{\frac{-x}{t_{1}}}+A_{2}\cdot e^{\frac{-x}{t_{2}}}+y_{0}$ (eq.S2)

A_i_ (i=1,2) are the amplitudes of the respective components, τ_i_ are the individual lifetime determined as τ_i_=t_i_*ln(2), when t_i_ means time constant (see Fig. S3).


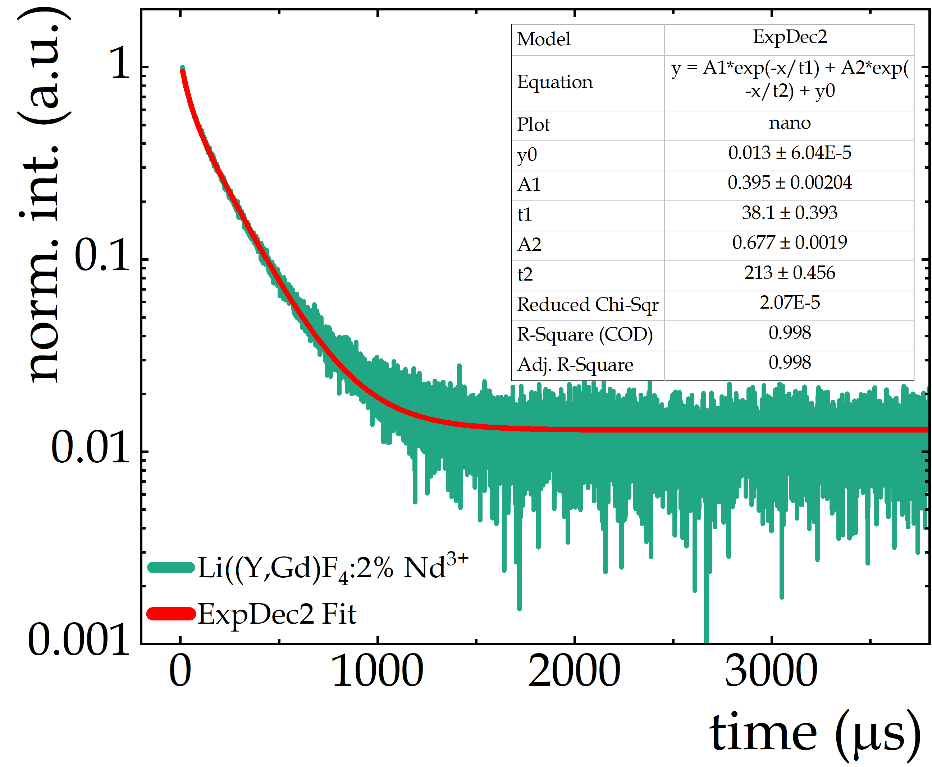


**Figure S3**. Decay curve and fit curve of the ExpDec2 function of an example Li(Y,Gd)F_4_:2%Nd^3+^ sample. The table shows the parameter values used to calculate the average time with the double-exponential function according to Equation S1.


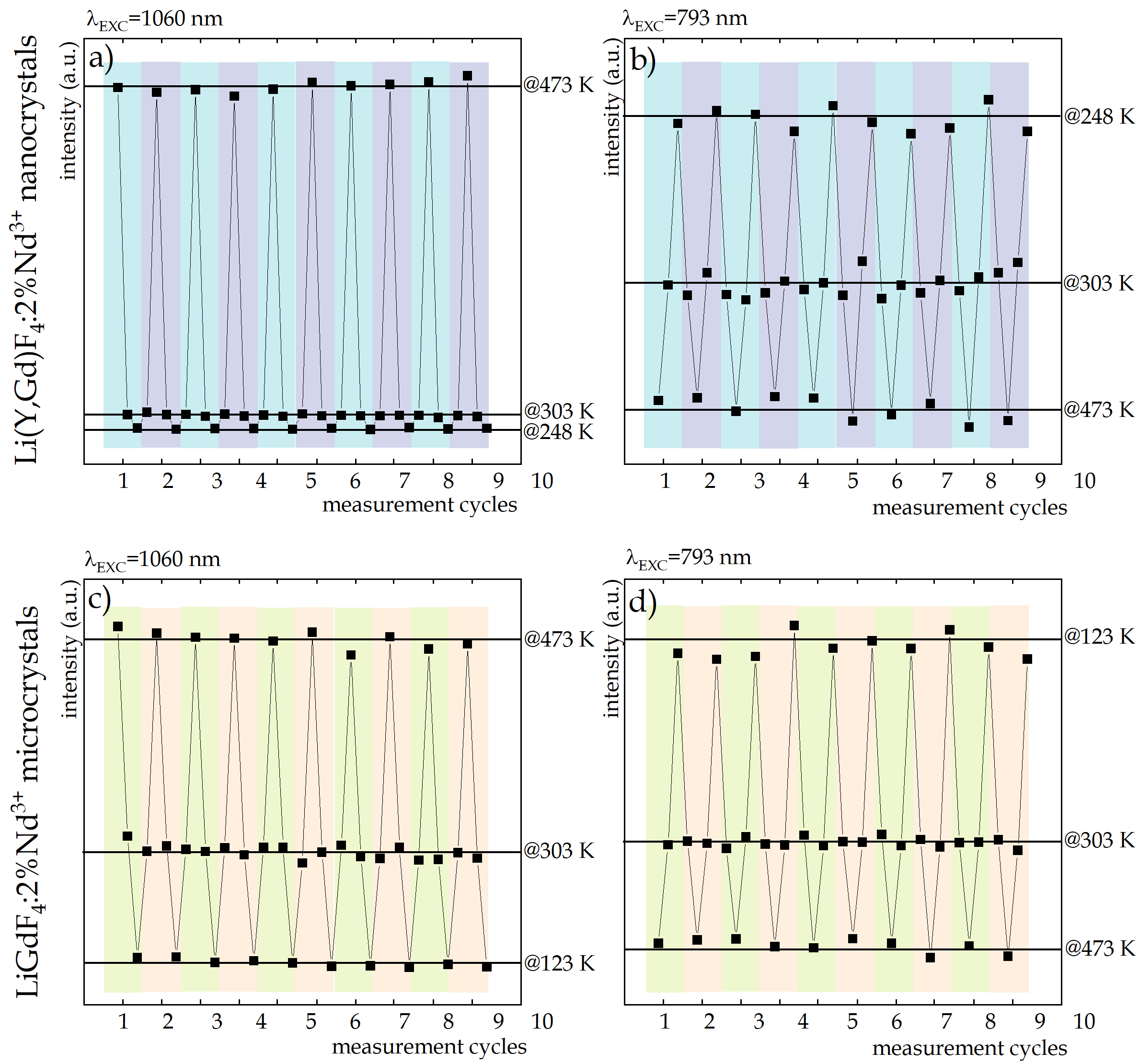


**Figure S4**. Changes in luminescence intensity over 10 heating and cooling cycles between temperature extremes, with an additional stop at the intermediate temperature of 303 K, obtained for representative Li(Y,Gd)F_4_:2%Nd^3+^ nanocrystals (a,b) and microcrystals (c,d) for both excitation sources.
